# Rigidez Arterial, Desfechos Clínico-Funcionais e Qualidade de Vida em Pacientes Pós-Covid-19: Um Estudo Transversal

**DOI:** 10.36660/abc.20250450

**Published:** 2026-07-16

**Authors:** Bruna Cavon Luna, Ana Cristina Lamezon, Emilton Lima, Lêda Maria Rabelo, Silvia Valderramas

**Affiliations:** 1 Programa de Pós-graduação em Medicina Interna e Ciências da Saúde Universidade Federal do Paraná Curitiba PR Brasil Programa de Pós-graduação em Medicina Interna e Ciências da Saúde da Universidade Federal do Paraná, Curitiba, PR – Brasil; 2 Hospital de Clínicas Universidade Federal do Paraná Curitiba PR Brasil Serviço de Pneumologia, Hospital de Clínicas, Universidade Federal do Paraná, Curitiba, PR - Brasil

**Keywords:** Rigidez Vascular, COVID-19, Hemodinâmica, Desempenho Físico Funcional

## Abstract

**Fundamento:**

O remodelamento arterial que culmina no aumento da rigidez arterial envolve mecanismos como estresse oxidativo, produção de espécies reativas de oxigênio, alterações neuroendócrinas e predisposição genética. Esses processos também estão presentes na fisiopatologia da covid-19. Entretanto, a relação entre rigidez arterial, parâmetros hemodinâmicos e desfechos clínico-funcionais em pacientes pós-covid-19 ainda não foi adequadamente investigada.

**Objetivos:**

Investigar a rigidez arterial, os parâmetros hemodinâmicos centrais e periféricos, o desempenho funcional, a qualidade de vida, a fadiga, a dispneia e a qualidade do sono em pacientes pós-covid-19.

**Métodos:**

Estudo transversal conduzido com indivíduos do grupo pós-covid-19 (GpC) e indivíduos pareados do grupo controle (n = 32). A rigidez arterial foi avaliada por meio da velocidade da onda de pulso (VOP) e do índice de aumentação corrigido para frequência cardíaca de 75 bpm (AIx@75). Foram avaliados o desempenho funcional pelo teste de sentar e levantar cinco vezes, a força de preensão manual, a qualidade de vida (avaliada pelo 12-Item Short Form Survey), a fadiga (avaliada pela Fatigue Severity Scale – versão brasileira), a dispneia (avaliada pela escala modificada do Medical Research Council) e a qualidade do sono.

**Resultados:**

O GpC apresentou valores mais elevados de VOP (Δ = 0,80 m/s; intervalo de confiança de 95% [IC95%], 0,08 a 1,52; p = 0,03) e AIx@75 (Δ = 8,34; IC95%, 2,02 a 14,66; p = 0,01), além de níveis mais elevados de pressão arterial central e periférica. Observou-se pior desempenho funcional (Δ = 4,39 s; IC95%, 2,70 a 6,08; p < 0,01) e pior qualidade de vida no componente físico (Δ = −9,35; IC95%, −12,45 a −6,25; p < 0,01). Fadiga, dispneia e qualidade do sono também apresentaram resultados significativamente piores no GpC. Na análise ajustada, a VOP associou-se independentemente à idade e à pressão arterial sistólica, mas não à condição pós-covid-19.

**Conclusão:**

Pacientes pós-covid-19 apresentam maior rigidez arterial, alterações hemodinâmicas e pior desempenho clínico-funcional. Contudo, a rigidez arterial parece ser predominantemente determinada por fatores hemodinâmicos e pelo envelhecimento, e não pela condição pós-covid-19 de forma independente, sugerindo que o comprometimento funcional observado decorra de mecanismos multifatoriais.

## Graphical abstract

**Figura Central:**
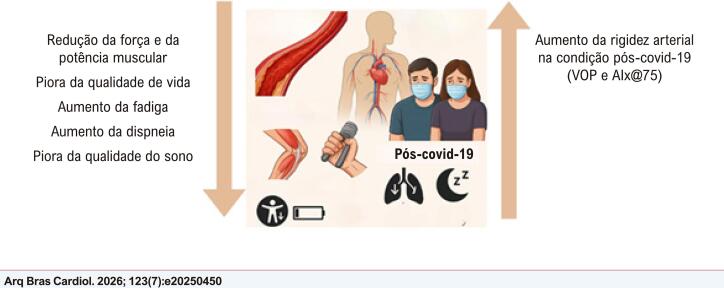
Rigidez Arterial, Desfechos Clínico-Funcionais e Qualidade de Vida em Pacientes Pós-Covid-19: Um Estudo Transversal

AIx@75: índice de aumentação corrigido para frequência cardíaca de 75 bpm; VOP: velocidade da onda de pulso.

## Introdução

A covid-19 é uma doença multissistêmica, e a maioria dos indivíduos acometidos apresenta recuperação completa após a fase aguda da infecção.^1^ No entanto, aproximadamente 10%-20% dos pacientes persistem com sintomas e alterações clínicas de médio e longo prazo, condição atualmente reconhecida como condição pós-covid-19 ou covid longa.^2^

A rigidez arterial, reconhecida como um importante marcador de risco cardiovascular, ocorre naturalmente como consequência do envelhecimento biológico. Entretanto, evidências recentes indicam que níveis elevados de rigidez arterial podem estar associados a diversos desfechos clínicos adversos, especialmente em populações com estados inflamatórios persistentes, como sobreviventes da covid-19.^3-5^

O aumento da rigidez arterial compromete a perfusão tecidual e está associado à disfunção endotelial e à inflamação sistêmica, fatores que podem contribuir para fadiga persistente, redução da capacidade funcional e piora da qualidade de vida.^5,6^ Além disso, alterações da hemodinâmica central podem interferir no controle ventilatório e na oxigenação tecidual, exacerbando sintomas como dispneia e distúrbios do sono.^5^ Por isso, é relevante investigar a possível relação entre a rigidez arterial e esses desfechos multidimensionais em pacientes pós-covid-19, visando ampliar a compreensão dos mecanismos fisiopatológicos envolvidos e subsidiar o desenvolvimento de estratégias terapêuticas mais eficazes.

A disfunção endotelial persistente e a inflamação vascular decorrentes da infecção pelo SARS-CoV-2 têm sido apontadas como mecanismos centrais na associação entre a covid-19 e complicações cardiovasculares de longo prazo, incluindo o aumento da rigidez arterial.

Nossa hipótese é que pacientes pós-covid-19 apresentam maior rigidez arterial, alterações dos parâmetros hemodinâmicos centrais e periféricos, pior desempenho funcional e qualidade de vida, além de maiores níveis de fadiga e dispneia, quando comparados a indivíduos saudáveis pareados. Para testar essa hipótese, foi conduzido um estudo com delineamento transversal.

Dessa forma, o objetivo deste estudo foi investigar a rigidez arterial, os parâmetros hemodinâmicos centrais e periféricos, o desempenho funcional, a qualidade de vida, a fadiga, a dispneia e a qualidade do sono em pacientes pós-covid-19.

## Métodos

### Delineamento do estudo e aspectos éticos

Trata-se de um estudo observacional transversal elaborado de acordo com as recomendações do STrengthening the Reporting of OBservational studies in Epidemiology^7^ e aprovado pelo comitê de ética em pesquisa com seres humanos da instituição.

### Participantes

A triagem dos participantes e a coleta de dados foram realizadas em um ambulatório de pneumologia. Foram incluídos indivíduos de ambos os sexos, com idade entre 40 e 75 anos, diagnóstico prévio de covid-19 confirmado por reação em cadeia da polimerase com transcrição reversa e condição pós-covid-19 estabelecida após pelo menos 12 semanas do início dos sintomas agudos. Os participantes deveriam ter apresentado quadros classificados como moderados, graves ou críticos^8^ e estar em acompanhamento no referido ambulatório.

O grupo controle (GC) foi composto por indivíduos saudáveis da comunidade. Caso tivessem histórico prévio de covid-19, a infecção deveria ter sido assintomática ou leve, sem necessidade de internação hospitalar, além da ausência de sintomas gripais nos 30 dias anteriores à avaliação. Os participantes foram pareados por sexo, idade, índice de massa corporal (IMC), comorbidades e Índice de Comorbidade de Charlson (ICC).^9^ O ICC é um instrumento utilizado para quantificar a gravidade das comorbidades por meio dos diagnósticos secundários e estimar seu impacto prognóstico, incluindo o risco de mortalidade.

Adicionalmente, todos os participantes deveriam apresentar escore no Mini Exame do Estado Mental (MEEM)^10^ ≥ 18 para indivíduos alfabetizados e ≥ 13 para indivíduos analfabetos, bem como independência para a realização das atividades instrumentais da vida diária.

Foram excluídos indivíduos com doença pulmonar obstrutiva crônica, asma, doença arterial coronariana (história prévia de infarto agudo do miocárdio ou revascularização), insuficiência cardíaca previamente diagnosticada, hipertensão arterial sistêmica (HAS) não controlada, diabetes melito, doença renal crônica (definida como taxa de filtração glomerular estimada < 60 ml/min/1,73 m^2^ por período superior a 3 meses), acidente vascular encefálico prévio, doenças neuromusculares ou degenerativas, uso de próteses ou órteses em membros inferiores que pudessem comprometer a realização dos testes funcionais, bem como indivíduos que estivessem participando ou tivessem participado de programas de condicionamento físico nos 3 meses anteriores.

### Recrutamento e coleta de dados

A seleção dos participantes foi realizada com base nos critérios de inclusão e exclusão previamente estabelecidos. O recrutamento do GC ocorreu por meio de ampla divulgação da pesquisa em ambiente hospitalar, incluindo funcionários e familiares, redes sociais e contatos pessoais das pesquisadoras.

Após esclarecimentos sobre os objetivos, procedimentos, riscos e benefícios do estudo, todos os participantes assinaram o Termo de Consentimento Livre e Esclarecido. Em seguida, foi realizada a coleta de dados por meio de ficha de avaliação elaborada pelos pesquisadores, contemplando informações demográficas, antropométricas e clínicas.

### Avaliação da rigidez arterial

A velocidade da onda de pulso (VOP), considerada o principal marcador de rigidez arterial, e o índice de aumentação corrigido para frequência cardíaca (FC) de 75 bpm (AIx@75), indicador da reflexão da onda de pulso influenciada pela rigidez arterial, foram avaliados de forma não invasiva utilizando o dispositivo Mobil-O-Graph^®^ (I.E.M. GmbH, Stolberg, Alemanha), previamente validado por Weiss et al.^11^

O equipamento utiliza um método oscilométrico baseado na aferição da pressão arterial braquial para estimar parâmetros centrais da circulação. A onda de pressão aórtica é reconstruída a partir da soma da onda incidente gerada pela ejeção ventricular e da onda refletida proveniente da periferia arterial.

A pressão de aumentação corresponde ao incremento da pressão arterial sistólica (PAS) central decorrente da reflexão da onda de pulso. Quando expressa como porcentagem da pressão de pulso central, origina o índice de aumentação. O AIx@75 é obtido mediante correção para FC de 75 bpm, realizada automaticamente pelo equipamento por meio de algoritmo previamente validado.

Foram obtidas três medidas consecutivas da onda de pulso aórtica, sendo utilizada a média dos valores para as análises.

A rigidez arterial aumentada foi definida como VOP > 8,2 m/s, de acordo com valores de referência estabelecidos em estudos que utilizaram o mesmo dispositivo empregado neste estudo.^12,13^

### Avaliação do desempenho funcional

O desempenho funcional foi avaliado por meio do teste de sentar e levantar cinco vezes (TSL5x)^14^ e da força de preensão manual (FPM).^15^

### Avaliação dos desfechos clínicos e de qualidade de vida

A fadiga foi avaliada pela Fatigue Severity Scale – versão brasileira (FSS-BR),^16^ a dispneia pela escala modificada do Medical Research Council,^17^ a qualidade de vida pelo questionário 12-Item Short Form Survey (SF-12)^18^ e a qualidade do sono pelo Índice de Qualidade do Sono de Pittsburgh.^19^

### Padronização dos procedimentos

Os testes funcionais seguiram uma sequência padronizada, incluindo uma tentativa de familiarização para cada procedimento e intervalo de repouso de 5 minutos entre as avaliações.

Como medida de segurança e para registro de possíveis eventos adversos, a FC, a frequência respiratória e a SpO_2_ foram monitoradas continuamente durante a realização dos testes.

Com o objetivo de garantir a padronização das avaliações e minimizar potenciais vieses de mensuração, todos os procedimentos foram conduzidos por uma única pesquisadora previamente treinada e capacitada.

### Desfechos do estudo

O desfecho primário do estudo foi a rigidez arterial, avaliada pela VOP. Os desfechos secundários incluíram os parâmetros hemodinâmicos centrais e periféricos, o desempenho funcional, a qualidade de vida, a fadiga, a dispneia e a qualidade do sono.

### Cálculo amostral

O cálculo amostral foi baseado na VOP, considerada o desfecho principal do estudo. Para essa estimativa, foram utilizados os resultados de Jud et al., que observaram valores de VOP de 10,75 ± 8,10 m/s em pacientes com covid-19 e de 5,30 ± 5,38 m/s nos respectivos controles.^20^

Considerando poder estatístico de 80%, erro tipo I (α = 0,05) e erro tipo II (β = 0,20), estimou-se a necessidade de 25 participantes por grupo para detectar diferenças significativas entre os grupos. Com o objetivo de compensar possíveis perdas, foi acrescido 22% ao tamanho amostral inicialmente calculado, resultando em uma amostra final de 64 participantes, distribuídos igualmente entre os grupos (32 participantes por grupo).

O cálculo amostral foi realizado utilizando o *software* G*Power versão 3.1^®^.

### Análise estatística

As análises estatísticas foram realizadas no *software* IBM SPSS Statistics for Windows, versão 22.0 (IBM Corp., Armonk, N.Y., EUA).

A normalidade dos dados foi avaliada pelo teste de Shapiro-Wilk e a homogeneidade das variâncias pelo teste de Levene. As variáveis contínuas foram apresentadas como média ± desvio padrão ou mediana (intervalo interquartil), conforme sua distribuição. As variáveis categóricas foram expressas em frequências absolutas e relativas.

As comparações entre o grupo pós-covid-19 (GpC) e o GC foram realizadas por meio do teste *t* de Student para amostras independentes, para variáveis com distribuição normal, do teste de Mann-Whitney para variáveis não paramétricas e dos testes do qui-quadrado ou exato de Fisher para variáveis categóricas.

Para investigar os fatores associados à VOP, foi realizada regressão linear múltipla, considerando a VOP como variável dependente e ajustando-se para sexo, idade, PAS e pressão arterial diastólica (PAD).

Os tamanhos de efeito foram expressos por meio da diferença padronizada de médias (d de Cohen) e da diferença absoluta entre médias, acompanhadas dos respectivos intervalos de confiança de 95% (IC95%). O nível de significância foi estabelecido em 5% (p < 0,05) para todas as análises.

## Resultados

**Figura 1:**
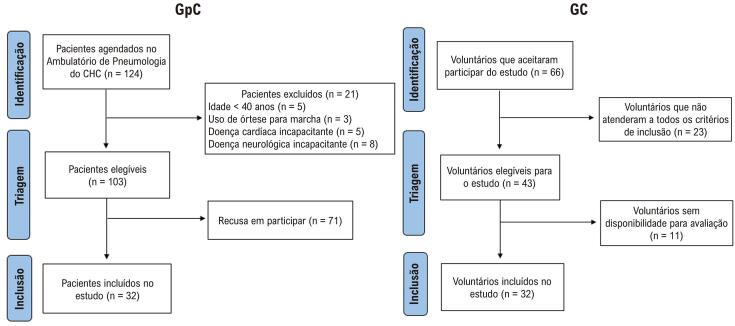
– Fluxograma de recrutamento, seleção e inclusão dos participantes do estudo. CHC: Complexo Hospital de Clinicas; GC: grupo controle; GpC: grupo pós-covid-19.

Durante o período de coleta, 124 pacientes potencialmente elegíveis foram recrutados para o estudo, dos quais 32 atenderam aos critérios de inclusão e foram alocados no GpC. Para o GC, 66 voluntários manifestaram interesse em participar da pesquisa, sendo 32 selecionados após pareamento por sexo, idade, IMC e comorbidades (Figura 1).

Foram avaliados 64 participantes, distribuídos igualmente entre o GpC (n = 32) e o GC (n = 32). Os grupos apresentaram características semelhantes quanto à idade, sexo, IMC e prevalência de HAS. Entretanto, o GpC apresentou pior desempenho cognitivo avaliado pelo MEEM em comparação ao GC (p < 0,01). Não foram observadas diferenças entre os grupos quanto à prática de atividade física e ao ICC. A distribuição das principais classes de medicamentos em uso também foi semelhante entre os grupos. As demais características da amostra estão apresentadas na Tabela 1.

**Tabela 1:** – Características demográficas, antropométricas e clínicas da amostra

Variáveis	GpC (n = 32)	GC (n = 32)	Valor de p
**Idade, anos**	58,80 ± 10,20	59,18 ± 9,62	0,87
**Sexo, M/F**	17/15	17/15	1,00
**IMC, kg/m**^**2**^	31,40 ± 5,10	29,20 ± 4,09	0,06
**Obesidade, IMC ≥ 30 kg/m**^**2**^	19 (59,40)	16 (46,90)	0,31
**HAS**	28 (87,50)	28 (87,50)	1,00
**Estado cognitivo, MEEM**	25,84 ± 4,11	28,71 ± 1,25	< 0,01
**Prática de atividade física, ≥ 150 min/semana**	15 (46,90)	13 (40,60)	0,86
**Tempo total de internação, dias**	25,50 ± 6,23	—	—
**Uso de oxigenioterapia**	26 (81,30)	—	—
**Uso de ventilação mecânica**	14 (43,80)	—	—
**ICC**	2,00 [1,64 a 3,04]	1,00 [1,20 a 2,47]	0,24
Medicações em uso			
BRAs	18 (56,30)	12 (37,50)	0,14
BCCs	14 (43,80)	15 (46,90)	0,80
Fármacos adrenérgicos	9 (28,10)	10 (31,30)	0,79
Diuréticos	19 (59,40)	18 (56,30)	0,80
Estatinas	10 (31,30)	9 (28,10)	0,79
Corticoides	12 (37,50)	6 (18,80)	0,10
Aspirina	20 (62,50)	22 (68,80)	0,60
Anticoagulantes	16 (50,00)	10 (31,30)	0,13
Antidepressivos e ansiolíticos	10 (31,30)	9 (28,10)	0,79
Hipoglicemiantes	10 (31,30)	10 (31,30)	1,00

*Dados apresentados como média ± desvio padrão, n (%) ou mediana (intervalo interquartil [percentis 25 a 75]), conforme apropriado. BCCs: bloqueadores dos canais de cálcio; BRAs: bloqueadores dos receptores da angiotensina II; GC: grupo controle; GpC: grupo pós-covid-19; HAS: hipertensão arterial sistêmica; ICC: Índice de Comorbidade de Charlson; IMC: índice de massa corporal; MEEM: Mini Exame do Estado Mental.*

A Tabela 2 apresenta os parâmetros de rigidez arterial e hemodinâmicos avaliados. Os participantes do GpC apresentaram valores significativamente mais elevados de VOP, AIx@75, PAS periférica, PAS central, PAD central e PAD periférica em comparação ao GC.

**Tabela 2:** – VOP, AIx@75 e variáveis hemodinâmicas

Variáveis	GpC (n = 32)	GC (n = 32)	Valor de p	d de Cohen	Diferença de médias (IC95%)
**VOP, m/s**	8,65 ± 1,56	7,85 ± 1,30	0,03	0,55	0,80 (0,08 a 1,52)
**AIx@75**	24,87 ± 13,20	16,53 ± 11,88	0,01	0,67	8,34 (2,02 a 14,66)
**PAS periférica, mmHg**	132,00 ± 15,57	122,00 ± 10,41	< 0,01	0,75	10,00 (3,90 a 16,10)
**PAD periférica, mmHg**	86,00 ± 10,90	81,00 ± 10,70	0,09	0,46	5,00 (−0,80 a 10,80)
**PAS central, mmHg**	122,00 ± 13,15	113,00 ± 9,36	< 0,01	0,79	9,00 (3,80 a 14,20)
**PAD central, mmHg**	86,00 ± 11,31	81,00 ± 10,01	0,04	0,47	5,00 (0,20 a 9,80)
**FC, bpm**	80,00 ± 13,09	73,00 ± 9,23	0,01	0,62	7,00 (2,00 a 12,00)

*Dados apresentados como média ± desvio padrão. AIx@75: índice de aumentação corrigido para frequência cardíaca de 75 bpm; FC: frequência cardíaca; GC: grupo controle; GpC: grupo pós-covid-19; IC95%: intervalo de confiança de 95%; PAD: pressão arterial diastólica; PAS: pressão arterial; VOP: velocidade da onda de pulso.*

Observou-se aumento significativo da VOP no GpC, com diferença média de 0,80 m/s (IC95%, 0,08 a 1,52; d = 0,55). Resultados semelhantes foram observados para o AIx@75 e para os parâmetros hemodinâmicos, com tamanhos de efeito variando de moderados a elevados.

Os desfechos clínico-funcionais estão apresentados na Tabela 3. O GpC apresentou pior desempenho funcional no TSL5x) (Δ = 4,39 s; IC95%, 2,70 a 6,08; d = 1,28) e menor qualidade de vida no componente físico do SF-12 (Δ = −9,35; IC95%, −12,45 a −6,25; d = −1,31). Além disso, foram observados maiores níveis de fadiga, dispneia e pior qualidade do sono no GpC, com tamanhos de efeito moderados a elevados.

**Tabela 3:** – Desempenho funcional, qualidade de vida, fadiga, dispneia e qualidade do sono dos grupos

Variáveis	GpC (n = 32)	GC (n = 32)	Valor de p	d de Cohen	Diferença de médias (IC95%)
Desempenho funcional					
TSL5x, s	15,09 ± 4,66	10,70 ± 1,38	< 0,01	1,28	4,39 (2,70 a 6,08)
FPM, kgf	30,93 ± 9,41	35,54 ± 9,14	0,05	0,16	–4,61 (–2,80 a 5,80)
Qualidade de vida, SF-12					
Componente físico	38,91 ± 8,74	48,26 ± 4,73	< 0,01	−1,31	–9,35 (–12,45 a –6,25)
Componente mental	44,49 ± 11,04	49,90 ± 6,20	0,03	−0,60	–5,41 (–10,40 a –0,42)
Fadiga, FSS-BR	4,65 [3,40 a 5,67]	1,00 [1,00 a 1,15]	< 0,01	2,45	3,65 (2,95 a 4,35)
Dispneia, mMRC	3,00 [2,00 a 3,00]	2,00 [1,00 a 2,00]	< 0,01	0,92	1,00 (0,45 a 1,55)
Qualidade do sono, PSQI	7,50 [5,00 a 12,00]	5,00 [3,25 a 6,00]	0,01	1,02	2,50 (1,30 a 3,70)

*Dados apresentados como média ± desvio padrão ou mediana (intervalo interquartil [percentis 25-75]), conforme apropriado. FPM: força de preensão manual; FSS-BR: Fatigue Severity Scale – versão brasileira; GC: grupo controle; GpC: grupo pós-covid-19; IC95%: intervalo de confiança de 95%; mMRC: escala modificada do Medical Research Council; PSQI: Índice de Qualidade do Sono de Pittsburgh; SF-12: 12-Item Short Form Health Survey; TSL5x: teste de sentar e levantar cinco vezes.*

Na análise de regressão linear múltipla, considerando a VOP como variável dependente e ajustando-se para sexo, idade, PAD e PAD, a associação entre VOP e condição pós-covid-19 não permaneceu significativa (β = 0,24; IC95%, −0,03 a 0,51; p = 0,08). Por outro lado, a idade (β = 0,117; IC95%, 0,104 a 0,130; p < 0,001) e a PAS (β = 0,032; IC95%, 0,026 a 0,039; p < 0,001) permaneceram independentemente associadas ao aumento da VOP.

Na análise de subgrupo, indivíduos com rigidez arterial (n = 18) apresentaram idade significativamente mais elevada e maiores níveis de pressão arterial periférica e central, com tamanhos de efeito moderados a elevados. Em contrapartida, não foram observadas diferenças significativas nos desfechos clínico-funcionais, incluindo desempenho funcional, fadiga, dispneia e qualidade de vida, cujos tamanhos de efeito foram pequenos e os IC95% incluíram o valor nulo (Tabela 4). O sexo e a PAD não apresentaram associação significativa com a VOP.

**Tabela 4:** – Características demográficas, antropométricas, hemodinâmicas e desfechos clínico-funcionais em pacientes pós-covid-19 com e sem rigidez arterial

Variáveis	Com rigidez arterial (n = 18)	Sem rigidez arterial (n = 14)	Valor de p	d de Cohen	Diferença de médias (IC95%)
**Idade, anos**	67,30 ± 5,10	49,60 ± 6,50	< 0,001	3,03	17,70 (13,50 a 21,90)
**Sexo, M/F**	5/13	4/10	0,910	—	—
**IMC, kg/m**^**2**^	29,90 ± 4,30	33,50 ± 5,80	0,080	–0,70	–3,60 (–7,80 a 0,60)
**VOP, m/s**	9,84 ± 0,85	7,12 ± 0,64	< 0,001	3,60	2,72 (2,20 a 3,24)
**PAS periférica, mmHg**	140,30 ± 15,20	126,40 ± 11,90	0,010	1,01	13,90 (4,20 a 23,60)
**PAD periférica, mmHg**	89,50 ± 10,80	83,60 ± 8,90	0,040	0,59	5,90 (0,40 a 11,40)
**PAS central, mmHg**	128,90 ± 14,10	116,80 ± 12,30	0,020	0,91	12,10 (3,00 a 21,20)
**PAD central, mmHg**	90,20 ± 11,50	84,70 ± 9,40	0,040	0,53	5,50 (0,30 a 10,70)
Desempenho funcional					
TSL5x, s	14,90 ± 4,20	16,30 ± 5,00	0,070	–0,01	-0,05 (–3,50 a 3,39)
FPM, kgf	32,60 ± 9,80	31,10 ± 8,70	0,220	–0,30	–1,40 (–4,80 a 2,00)
Qualidade de vida, SF-12					
Componente físico	39,70 ± 8,90	38,80 ± 9,10	0,330	0,16	1,50 (–5,30 a 8,30)
Componente mental	41,20 ± 10,50	44,50 ± 11,20	0,290	–0,10	0,90 (–5,80 a 7,60)
**Fadiga, FSS-BR**	4,60 [3,30 a 5,30]	4,90 [4,20 a 5,70]	0,310	–0,30	–0,30 (–1,20 a 0,60)
**Dispneia, mMRC**	3,00 [2,00 a 3,00]	2,00 [2,00 a 3,00]	0,410	–0,20	–0,62 (–1,71 a 0,46)
**Qualidade do sono, PSQI**	7,00 [5,00 a 10,00]	8,00 [6,00 a 12,00]	0,380	0,25	–1,00 (–0,50 a 2,50)

*Dados apresentados como média ± desvio padrão ou mediana (intervalo interquartil [percentis 25-75]), conforme apropriado. FPM: força de preensão manual; FSS-BR: Fatigue Severity Scale – versão brasileira; IC95%: intervalo de confiança de 95%; IMC: índice de massa corporal; mMRC: escala modificada do Medical Research Council; PAD: pressão arterial diastólica; PAS: pressão arterial sistólica; PSQI: Índice de Qualidade do Sono de Pittsburgh; SF-12: 12-Item Short Form Health Survey; TSL5x: teste de sentar e levantar cinco vezes; VOP: velocidade da onda de pulso.*

A Figura Central resume os principais achados do estudo, ilustrando o aumento da rigidez arterial e das alterações hemodinâmicas, bem como a piora do desempenho funcional, da fadiga, da dispneia, da qualidade de vida e da qualidade do sono observadas no GpC.

## Discussão

Os resultados deste estudo demonstraram que indivíduos pós-covid-19 apresentam aumento significativo da rigidez arterial, evidenciado por valores mais elevados de VOP e AIx@75, além de alterações hemodinâmicas centrais e periféricas. Adicionalmente, esses indivíduos apresentaram pior desempenho funcional, maiores níveis de fadiga e dispneia e comprometimento da qualidade de vida quando comparados a controles pareados por idade, sexo, IMC e comorbidades. Em conjunto, esses achados sugerem a persistência de alterações cardiovasculares e clínico-funcionais após a infecção pelo SARS-CoV-2, com potenciais implicações a médio e longo prazo.

Embora a faixa etária tenha sido definida por representar o perfil assistencial do serviço de pneumologia onde o estudo foi conduzido, a maioria dos participantes apresentava idade superior a 60 anos e excesso de peso ou obesidade. Esse achado é consistente com a literatura, que identifica idade avançada e obesidade como importantes fatores de risco para infecção por SARS-CoV-2 e para o desenvolvimento de formas graves da doença.^21,22^

Embora não tenha sido objeto de avaliação direta, o impacto psicológico associado à covid-19 mostrou-se relevante durante o recrutamento. Dos 103 pacientes elegíveis, 71 recusaram participação no estudo, relatando não desejar reviver experiências traumáticas relacionadas à doença ou às perdas familiares decorrentes da pandemia. Esse achado corrobora evidências que demonstram elevada frequência de sofrimento emocional, transtornos psicológicos e manifestações relacionadas ao trauma em sobreviventes da covid-19.^23,24^

Em relação às medicações em uso, observou-se predomínio de fármacos empregados no manejo de condições clínicas estáveis, incluindo anti-hipertensivos para HAS controlada (p.ex., inibidores da enzima conversora da angiotensina, bloqueadores dos receptores de angiotensina II, bloqueadores dos canais de cálcio e diuréticos), estatinas e psicofármacos (p.ex., inibidores seletivos da recaptação de serotonina e ansiolíticos), sem diferenças significativas entre os grupos. Dessa forma, a influência medicamentosa sobre os parâmetros de rigidez arterial e hemodinâmicos avaliados provavelmente foi limitada, reduzindo a possibilidade de viés de confusão relevante.

As alterações vasculares observadas podem estar relacionadas a mecanismos fisiopatológicos descritos na covid-19, incluindo disfunção endotelial persistente, inflamação crônica, ativação da coagulação e desregulação do sistema renina-angiotensina-aldosterona.^25-27^ Quando associadas a comorbidades pré-existentes, como HAS e obesidade, essas alterações podem potencializar o risco cardiovascular em médio e longo prazo.^28,29^

A associação entre o aumento da rigidez arterial e o pior desempenho funcional observada neste estudo sugere um possível papel da disfunção vascular na limitação funcional desses pacientes. A redução da complacência arterial pode comprometer a perfusão tecidual durante o esforço, contribuindo para maior percepção de fadiga e dispneia, mesmo na ausência de doença cardiovascular estabelecida.

No presente estudo, os indivíduos do GpC apresentaram aumento da rigidez arterial, acompanhado de níveis mais elevados de PAS periférica e central. Considerando a estreita relação fisiopatológica entre pressão arterial e rigidez arterial, não é possível determinar se a elevação da VOP contribuiu para os níveis pressóricos mais elevados, se a pressão arterial influenciou os valores da VOP ou se ambas compartilham determinantes fisiopatológicos comuns. Essa limitação é inerente ao delineamento transversal e reforça a necessidade de estudos longitudinais capazes de esclarecer a direção temporal dessas associações.

Adicionalmente, na análise de regressão linear múltipla ajustada para idade, sexo e níveis pressóricos, a associação entre a condição pós-covid-19 e os valores de VOP não permaneceu estatisticamente significativa. Em contrapartida, a idade e a PAS mantiveram associação independente com o aumento da VOP. Esses achados sugerem que a maior rigidez arterial observada no GpC pode ser explicada predominantemente por fatores hemodinâmicos e pelo envelhecimento vascular, mais do que por um efeito independente da condição pós-covid-19.

Esse resultado reforça a natureza multifatorial da rigidez arterial e está em consonância com a literatura, que reconhece a idade e a pressão arterial como seus principais determinantes fisiológicos. No contexto da covid-19, embora mecanismos como disfunção endotelial e inflamação persistente tenham sido amplamente descritos, os resultados do presente estudo sugerem que essas alterações podem atuar como fatores potencializadores sobre um substrato vascular previamente vulnerável, e não necessariamente como determinantes independentes da rigidez arterial.

Adicionalmente, a ausência de associação independente após o ajuste reforça a necessidade de cautela na interpretação de análises baseadas na categorização da VOP, uma vez que a própria definição de rigidez arterial foi estabelecida a partir desse parâmetro. Nesse contexto, a utilização da VOP como variável contínua pode representar uma abordagem metodologicamente mais robusta em estudos futuros.

Nesse contexto, a análise de subgrupo comparando pacientes pós-covid-19 com e sem rigidez arterial (Tabela 4) mostrou-se particularmente esclarecedora: apesar da idade significativamente mais elevada e dos maiores níveis pressóricos centrais e periféricos observados no subgrupo com rigidez arterial, não foram identificadas diferenças significativas nos desfechos clínico-funcionais — incluindo desempenho funcional, fadiga, dispneia e qualidade de vida — entre os subgrupos, com tamanhos de efeito pequenos e intervalos de confiança de 95% incluindo o valor nulo. Esses resultados devem ser interpretados com cautela, dado o tamanho amostral reduzido da análise de subgrupo, que pode ter limitado o poder estatístico para detectar diferenças reais.

Essa aparente dissociação sugere que, embora a VOP esteja fortemente associada ao envelhecimento vascular e a determinantes hemodinâmicos, sua presença isolada não explica integralmente o comprometimento funcional observado na condição pós-covid-19. De fato, os pacientes do GpC apresentaram pior desempenho funcional, maiores níveis de fadiga e dispneia e pior qualidade de vida em comparação ao GC; esses achados podem ser mais bem explicados por mecanismos adicionais — incluindo inflamação sistêmica persistente, internação prolongada, inatividade física, perda de massa muscular e alterações musculoesqueléticas — atuando de forma independente ou sinérgica às alterações vasculares observadas, e não necessariamente pela rigidez arterial isoladamente.^30,31^

Os pacientes do GpC apresentaram pior desempenho funcional, maiores níveis de fadiga e dispneia e pior qualidade de vida em comparação ao GC. Esses achados podem ser explicados por fatores como inflamação sistêmica persistente, internação prolongada, inatividade física, perda de massa muscular e falta de condicionamento cardiorrespiratório.^30,31^

Nossos resultados são consistentes com estudos prévios que demonstram comprometimento funcional persistente após a fase aguda da covid-19. Em uma coorte previamente publicada, os participantes foram classificados em quatro grupos de acordo com o grau de comprometimento físico e mental, sendo observada prevalência de comprometimento grave em 21% dos indivíduos e muito grave em 17%. Além disso, 46,2% apresentavam comprometimento funcional persistente, mesmo quando avaliados por instrumentos distintos daqueles utilizados neste estudo.^32^

De forma semelhante, Huang et al. acompanharam pacientes por 12 meses após a infecção e observaram maior prevalência de sintomas persistentes em comparação aos controles, incluindo dispneia, limitações de mobilidade, ansiedade, depressão e fadiga, especialmente entre as mulheres.^33^

Resultados semelhantes foram descritos por Seeßle et al., que identificaram redução da capacidade de exercício, fadiga persistente e distúrbios do sono em mais da metade dos pacientes avaliados 12 meses após a infecção.^34^ Outros estudos também apontam para comprometimentos físicos e mentais prolongados, associados à maior gravidade da doença durante a fase aguda da covid-19.

Estudos intervencionistas também têm demonstrado benefícios terapêuticos relevantes nessa população. Em ensaio clínico conduzido por Jimeno-Almazán et al., 82% dos participantes relataram fadiga, 59% dispneia e 51,3% distúrbios do sono. Após oito semanas de treinamento supervisionado, observou-se melhora significativa da fadiga avaliada pela FSS-BR, da qualidade de vida avaliada pelo SF-12, da percepção de dispneia, da FPM e do desempenho funcional avaliado pelo TSL5x.^35^

De forma consistente, programas estruturados de reabilitação têm demonstrado benefícios sobre capacidade funcional, força muscular, fadiga, qualidade do sono e dispneia, reforçando a importância de abordagens terapêuticas multidisciplinares para indivíduos com sintomas persistentes após a covid-19.^36-38^

Considerando os resultados observados e o reconhecimento crescente da relevância clínica da rigidez arterial como marcador cardiovascular, reforça-se a importância da avaliação abrangente e precoce de pacientes pós-covid-19, especialmente daqueles com fatores de risco cardiovascular. A identificação de alterações vasculares, hemodinâmicas, funcionais e psicossociais pode contribuir para o direcionamento de estratégias de monitoramento e reabilitação. Embora a avaliação da rigidez arterial ainda seja relativamente pouco utilizada na prática clínica brasileira, trata-se de um método não invasivo, reprodutível e de baixo custo, com potencial aplicação nesse contexto.

### Limitações do estuo

Este estudo apresenta algumas limitações. O delineamento transversal impede a inferência de causalidade entre a infecção por SARS-CoV-2 e as alterações observadas, não sendo possível estabelecer a direção temporal dessas associações. Além disso, variáveis potencialmente relevantes para a rigidez arterial, como perfil lipídico, padrão alimentar e histórico familiar de doença cardiovascular, não foram avaliadas. A elevada taxa de recusa durante o recrutamento pode ter introduzido viés de seleção, uma vez que indivíduos com maior impacto psicológico podem ter sido menos propensos a participar do estudo. Por fim, embora validado, o dispositivo oscilométrico utilizado não corresponde ao método tonométrico considerado padrão de referência para avaliação da rigidez arterial.

Por outro lado, segundo o conhecimento dos autores, poucos estudos avaliaram simultaneamente a rigidez arterial, os parâmetros hemodinâmicos e os desfechos clínico-funcionais em pacientes pós-covid-19, o que confere originalidade e relevância aos achados apresentados.

## Conclusão

Os pacientes pós-covid-19 avaliados neste estudo apresentaram aumento da rigidez arterial, alterações significativas do AIx@75 e das PAS central e periférica, além de pior desempenho funcional, pior qualidade de vida, maiores níveis de fadiga e dispneia e pior qualidade do sono. Esses achados reforçam a complexidade da condição pós-covid-19 e destacam a necessidade de estudos longitudinais para melhor compreensão dos mecanismos envolvidos. Adicionalmente, ressaltam a importância da avaliação cardiovascular e funcional desses pacientes, bem como da implementação de estratégias terapêuticas multidisciplinares voltadas ao seu acompanhamento e reabilitação.
