# Supplementary material for: Rigidez Arterial, Desfechos Clínico-Funcionais e Qualidade de Vida em Pacientes Pós-Covid-19: Um Estudo Transversal
Source: Arq Bras Cardiol. 2026 Jul 16;123(7):e20250450. [Article in Portuguese] doi: 10.36660/abc.20250450 (PMC13399114; doi:10.36660/abc.20250450)
Supplement: Translation

# Translation: Arterial Stiffness, Clinical-Functional Outcomes, and Quality of Life in Post-COVID-19 Patients: A Cross-Sectional Study

**DOI:** 10.36660/abc.20250450i

**Authors:** Bruna Cavon Luna, Ana Cristina Lamezon, Emilton Lima, Lêda Maria Rabelo, Silvia Valderramas

**Affiliations:** 1 Brazil Programa de Pós-graduação em Medicina Interna e Ciências da Saúde da Universidade Federal do Paraná, Curitiba, PR – Brazil; 2 Brazil Serviço de Pneumologia, Hospital de Clínicas, Universidade Federal do Paraná, Curitiba, PR - Brazil

**Keywords:** Vascular Stiffness, COVID-19, Hemodynamics, Physical Functional Performance

## Abstract

**Background:**

Arterial remodeling leading to increased arterial stiffness involves mechanisms such as oxidative stress, reactive oxygen species production, neuroendocrine alterations, and genetic predisposition. These processes are also involved in the pathophysiology of COVID-19. However, the relationship between arterial stiffness, hemodynamic parameters, and clinical-functional outcomes in post-COVID-19 patients has not yet been adequately investigated.

**Objectives:**

To investigate arterial stiffness, central and peripheral hemodynamic parameters, functional performance, quality of life, fatigue, dyspnea, and sleep quality in post-COVID-19 patients.

**Methods:**

This cross-sectional study was conducted with individuals in the post-COVID-19 group (PCG) and matched individuals in the control group (n = 32). Arterial stiffness was assessed using pulse wave velocity (PWV) and the augmentation index corrected to a heart rate of 75 bpm (AIx@75). Functional performance was evaluated using the five-times sit-to-stand test, handgrip strength, quality of life (assessed with the 12-Item Short Form Survey), fatigue (assessed with the Brazilian version of the Fatigue Severity Scale), dyspnea (assessed with the modified Medical Research Council scale), and sleep quality.

**Results:**

The PCG showed higher PWV values (Δ = 0.80 m/s; 95%CI, 0.08 to 1.52; p = 0.03) and AIx@75 (Δ = 8.34; 95%CI, 2.02 to 14.66; p = 0.01) as well as higher levels of central and peripheral blood pressure. Worse functional performance was observed (Δ = 4.39 s; 95%CI, 2.70 to 6.08; p < 0.01), along with poorer quality of life in the physical component (Δ = −9.35; 95%CI, −12.45 to −6.25; p < 0.01). Fatigue, dyspnea, and sleep quality outcomes were also significantly worse in the PCG. In the adjusted analysis, PWV was independently associated with age and systolic blood pressure, but not with post-COVID-19 status.

**Conclusion:**

Post-COVID-19 patients exhibit greater arterial stiffness, hemodynamic alterations, and poorer clinical-functional performance. However, arterial stiffness appears to be predominantly determined by hemodynamic factors and aging rather than by post-COVID-19 status independently, suggesting that the observed functional impairment results from multifactorial mechanisms.

## Introduction

COVID-19 is a multisystem disease, and most affected individuals experience complete recovery after the acute phase of infection.^1^ However, approximately 10%-20% of patients continue to experience persistent symptoms and medium- to long-term clinical alterations, a condition currently recognized as post-COVID-19 condition or long COVID.^2^

Arterial stiffness, recognized as an important marker of cardiovascular risk, occurs naturally as a consequence of biological aging. However, recent evidence indicates that increased levels of arterial stiffness may be associated with several adverse clinical outcomes, particularly in populations with persistent inflammatory states, such as COVID-19 survivors.^3-5^

Increased arterial stiffness impairs tissue perfusion and is associated with endothelial dysfunction and systemic inflammation, factors that may contribute to persistent fatigue, decreased functional capacity, and poorer quality of life.^5,6^ Furthermore, alterations in central hemodynamics may interfere with ventilatory control and tissue oxygenation, exacerbating symptoms such as dyspnea and sleep disturbances.^5^ Therefore, investigating the potential relationship between arterial stiffness and these multidimensional outcomes in post-COVID-19 patients is relevant to improving the understanding of the underlying pathophysiological mechanisms and supporting the development of more effective therapeutic strategies.

Persistent endothelial dysfunction and vascular inflammation resulting from SARS-CoV-2 infection have been identified as central mechanisms linking COVID-19 to long-term cardiovascular complications, including increased arterial stiffness.

We hypothesized that post-COVID-19 patients would exhibit greater arterial stiffness, alterations in central and peripheral hemodynamic parameters, poorer functional performance and quality of life, and higher levels of fatigue and dyspnea compared with matched healthy individuals. To test this hypothesis, a cross-sectional study was conducted.

Accordingly, the aim of this study was to investigate arterial stiffness, central and peripheral hemodynamic parameters, functional performance, quality of life, fatigue, dyspnea, and sleep quality in post-COVID-19 patients.

## Methods

### Study design and ethical considerations

This was a cross-sectional observational study conducted in accordance with the STrengthening the Reporting of OBservational studies in Epidemiology recommendations^7^ and approved by the institution’s human research ethics committee.

### Participants

Participant screening and data collection were conducted at a pulmonology outpatient clinic. Eligible participants included men and women aged 40 to 75 years with a previous diagnosis of COVID-19 confirmed by reverse transcription polymerase chain reaction and a diagnosis of post-COVID-19 condition established at least 12 weeks after the onset of acute symptoms. Participants were required to have experienced moderate, severe, or critical disease^8^ and to be under follow-up at the outpatient clinic.

The control group (CG) consisted of healthy community-dwelling individuals. If they had a previous history of COVID-19, the infection must have been asymptomatic or mild, without requiring hospitalization, and they must have reported no flu-like symptoms within 30 days before the assessment. Participants were matched for sex, age, body mass index (BMI), comorbidities, and Charlson Comorbidity Index (CCI).^9^ The CCI is a tool used to quantify the severity of comorbidities based on secondary diagnoses and to estimate their prognostic impact, including mortality risk.

Additionally, all participants were required to have a Mini-Mental State Examination (MMSE)^10^ ≥ 18 for literate individuals and ≥ 13 for illiterate individuals, as well as independence in performing instrumental activities of daily living.

Individuals with chronic obstructive pulmonary disease, asthma, coronary artery disease (previous history of acute myocardial infarction or revascularization), previously diagnosed heart failure, uncontrolled hypertension, diabetes mellitus, chronic kidney disease (defined as an estimated glomerular filtration rate < 60 mL/min/1.73 m^2^ for more than 3 months), previous stroke, neuromuscular or degenerative diseases, use of lower-limb prostheses or orthoses that could interfere with functional testing, or participation in physical conditioning programs within the previous 3 months were excluded.

### Recruitment and data collection

Participants were selected according to the predefined inclusion and exclusion criteria. Recruitment of the CG was conducted through broad dissemination of the study within the hospital environment, including employees and family members, social media platforms, and the researchers’ personal contacts.

After receiving detailed information regarding the objectives, procedures, risks, and benefits of the study, all participants provided written informed consent. Subsequently, data were collected using an assessment form developed by the researchers, including demographic, anthropometric, and clinical information.

### Assessment of arterial stiffness

Pulse wave velocity (PWV), considered the primary marker of arterial stiffness, and the augmentation index corrected to a heart rate (HR) of 75 bpm (AIx@75), an indicator of pulse wave reflection influenced by arterial stiffness, were assessed noninvasively using the Mobil-O-Graph^®^ device (I.E.M. GmbH, Stolberg, Germany), previously validated by Weiss et al.^11^

The device uses an oscillometric method based on brachial blood pressure measurements to estimate central circulatory parameters. The aortic pressure waveform is reconstructed from the sum of the forward wave generated by ventricular ejection and the reflected wave originating from the peripheral arterial system.

Augmentation pressure corresponds to the increase in central systolic blood pressure (SBP) resulting from pulse wave reflection. When expressed as a percentage of central pulse pressure, it yields the augmentation index. AIx@75 is obtained through automatic correction to an HR of 75 bpm using a previously validated algorithm incorporated into the device.

Three consecutive measurements of the aortic pulse waveform were obtained, and the mean value was used for analysis.

Increased arterial stiffness was defined as PWV > 8.2 m/s according to reference values established in studies using the same device employed in the present study.^12,13^

### Assessment of functional performance

Functional performance was assessed using the five-times sit-to-stand test (5STS)^14^ and handgrip strength (HGS).^15^

### Assessment of clinical outcomes and quality of life

Fatigue was assessed using the Fatigue Severity Scale – Brazilian version (FSS-BR),^16^ dyspnea using the modified Medical Research Council scale,^17^ quality of life using the 12-Item Short Form Survey (SF-12),^18^ and sleep quality using the Pittsburgh Sleep Quality Index.^19^

### Standardization of procedures

Functional tests followed a standardized sequence, including one familiarization trial for each procedure and a 5-minute rest interval between assessments.

As a safety measure and to document potential adverse events, HR, respiratory rate, and SpO_2_ were continuously monitored throughout testing.

To ensure standardized assessments and minimize potential measurement bias, all procedures were conducted by a single trained and qualified researcher.

### Study outcomes

The primary outcome was arterial stiffness, assessed by PWV. Secondary outcomes included central and peripheral hemodynamic parameters, functional performance, quality of life, fatigue, dyspnea, and sleep quality.

### Sample size calculation

Sample size calculation was based on PWV, which was considered the primary outcome of the study. For this estimation, data from Jud et al. were used, who reported PWV values of 10.75 ± 8.10 m/s in patients with COVID-19 and 5.30 ± 5.38 m/s in their respective controls.^20^

Assuming 80% statistical power, a type I error (α = 0.05), and a type II error (β = 0.20), a minimum of 25 participants per group was required to detect significant differences between groups. To account for potential attrition, 22% was added to the initially calculated sample size, resulting in a final sample of 64 participants equally distributed between the groups (32 participants per group).

Sample size calculation was performed using G*Power software version 3.1^®^.

### Statistical analysis

Statistical analyses were performed using IBM SPSS Statistics for Windows, version 22.0 (IBM Corp., Armonk, N.Y., USA).

Data normality was assessed using the Shapiro-Wilk test, and homogeneity of variances was evaluated using Levene test. Continuous variables were presented as mean ± SD or median (IQR), according to data distribution. Categorical variables were expressed as absolute and relative frequencies.

Comparisons between the post-COVID-19 group (PCG) and the CG were performed using the independent-samples Student’s *t* test for normally distributed variables, the Mann-Whitney *U* test for nonparametric variables, and the chi-square test or Fisher’s exact test for categorical variables.

To investigate factors associated with PWV, multiple linear regression analysis was performed, with PWV as the dependent variable and adjustment for sex, age, SBP, and diastolic blood pressure (DBP).

Effect sizes were expressed as standardized mean differences (Cohen’s d) and absolute mean differences, accompanied by their respective 95%CIs. Statistical significance was set at 5% (p < 0.05) for all analyses.

## Results

During the data collection period, 124 potentially eligible patients were recruited for the study, of whom 32 met the inclusion criteria and were allocated to the PCG. For the CG, 66 volunteers expressed interest in participating, and 32 were selected after matching for sex, age, BMI, and comorbidities (Figure 1).

**Figure 1:** – Flowchart of participant recruitment, screening, and inclusion in the study. CG: control group; CHC: Complexo Hospital de Clinicas; PCG: post-COVID-19 group.

A total of 64 participants were evaluated, equally distributed between the PCG (n = 32) and the CG (n = 32). The groups were similar with respect to age, sex, BMI, and prevalence of systemic arterial hypertension. However, the PCG demonstrated poorer cognitive performance as assessed by the MMSE compared with the CG (p < 0.01). No differences were observed between groups regarding physical activity levels or CCI scores. The distribution of the main medication classes in use was also similar between groups. The remaining sample characteristics are presented in Table 1.

**Table 1:** – Demographic, anthropometric, and clinical characteristics of the sample

Variables	PCG (n = 32)	CG (n = 32)	p-value
**Age, years**	58.80 ± 10.20	59.18 ± 9.62	0.87
**Sex, M/F**	17/15	17/15	1.00
**BMI, kg/m**^**2**^	31.40 ± 5.10	29.20 ± 4.09	0.06
**Obesity, BMI ≥ 30 kg/m**^**2**^	19 (59.40)	16 (46.90)	0.31
**Hypertension**	28 (87.50)	28 (87.50)	1.00
**Cognitive status, MMSE**	25.84 ± 4.11	28.71 ± 1.25	< 0.01
**Physical activity, ≥ 150 min/week**	15 (46.90)	13 (40.60)	0.86
**Total length of hospitalization, days**	25.50 ± 6.23	—	—
**Use of oxygen therapy**	26 (81.30)	—	—
**Use of mechanical ventilation**	14 (43.80)	—	—
**CCI**	2.00 [1.64 to 3.04]	1.00 [1.20 to 2.47]	0.24
Medications in use			
ARBs	18 (56.30)	12 (37.50)	0.14
CCBs	14 (43.80)	15 (46.90)	0.80
Adrenergic agents	9 (28.10)	10 (31.30)	0.79
Diuretics	19 (59.40)	18 (56.30)	0.80
Statins	10 (31.30)	9 (28.10)	0.79
Corticosteroids	12 (37.50)	6 (18.80)	0.10
Aspirin	20 (62.50)	22 (68.80)	0.60
Anticoagulants	16 (50.00)	10 (31.30)	0.13
Antidepressants and anxiolytics	10 (31.30)	9 (28.10)	0.79
Hypoglycemic agents	10 (31.30)	10 (31.30)	1.00

Data are presented as mean ± SD, n (%), or median (IQR [25th-75th percentiles]), as appropriate. ARBs: angiotensin ii receptor blockers; BMI: body mass index; CCBs: calcium channel blockers; CCI: Charlson comorbidity index; CG: control group; MMSE: Mini-Mental State Examination; PCG: post-COVID-19 group.

Table 2 presents the arterial stiffness and hemodynamic parameters evaluated. Participants in the PCG exhibited significantly higher values of PWV, AIx@75, peripheral SBP, central SBP, central DBP, and peripheral DBP compared with the CG.

**Table 2:** – PWV, AIx@75, and hemodynamic variables

Variables	PCG (n = 32)	CG (n = 32)	p-value	Cohen’s d	Mean difference (95%CI)
**PWV, m/s**	8.65 ± 1.56	7.85 ± 1.30	0.03	0.55	0.80 (0.08 to 1.52)
**AIx@75**	24.87 ± 13.20	16.53 ± 11.88	0.01	0.67	8.34 (2.02 to 14.66)
**Peripheral SBP, mmHg**	132.00 ± 15.57	122.00 ± 10.41	< 0.01	0.75	10.00 (3.90 to 16.10)
**Peripheral DBP, mmHg**	86.00 ± 10.90	81.00 ± 10.70	0.09	0.46	5.00 (−0.80 to 10.80)
**Central SBP, mmHg**	122.00 ± 13.15	113.00 ± 9.36	< 0.01	0.79	9.00 (3.80 to 14.20)
**Central DBP, mmHg**	86.00 ± 11.31	81.00 ± 10.01	0.04	0.47	5.00 (0.20 to 9.80)
**HR, bpm**	80.00 ± 13.09	73.00 ± 9.23	0.01	0.62	7.00 (2.00 to 12.00)

Data are presented as mean ± SD. AIx@75: augmentation index corrected to a heart rate of 75 bpm; CG: control group; DBP: diastolic blood pressure; HR: heart rate; PCG: post-COVID-19 group; PWV: pulse wave velocity; SBP: systolic blood pressure.

A significant increase in PWV was observed in the PCG, with a mean difference of 0.80 m/s (95%CI, 0.08 to 1.52; d = 0.55). Similar findings were observed for AIx@75 and the hemodynamic parameters, with effect sizes ranging from moderate to large.

Clinical-functional outcomes are presented in Table 3. The PCG showed poorer functional performance on the 5STS (Δ = 4.39 s; 95%CI, 2.70 to 6.08; d = 1.28) and lower quality of life in the physical component of the SF-12 (Δ = −9.35; 95%CI, −12.45 to −6.25; d = −1.31). In addition, higher levels of fatigue, dyspnea, and poorer sleep quality were observed in the PCG, with moderate-to-large effect sizes.

**Table 3:** – Functional performance, quality of life, fatigue, dyspnea, and sleep quality in the study groups

Variables	PCG (n = 32)	CG (n = 32)	p-value	Cohen’s d	Mean difference (95%CI)
Functional performance					
5STS, s	15.09 ± 4.66	10.70 ± 1.38	< 0.01	1.28	4.39 (2.70 to 6.08)
HGS, kgf	30.93 ± 9.41	35.54 ± 9.14	0.05	0.16	– 4.61 (−2.80 to 5.80)
Quality of life, SF-12					
Physical component	38.91 ± 8.74	48.26 ± 4.73	< 0.01	−1.31	−9.35 (−12.45 to −6.25)
Mental component	44.49 ± 11.04	49.90 ± 6.20	0.03	−0.60	−5.41 (−10.40 to −0.42)
Fatigue, FSS-BR	4.65 [3.40 to 5.67]	1.00 [1.00 to 1.15]	< 0.01	2.45	3.65 (2.95 to 4.35)
Dyspnea, mMRC	3.00 [2.00 to 3.00]	2.00 [1.00 to 2.00]	< 0.01	0.92	1.00 (0.45 to 1.55)
Sleep quality, PSQI	7.50 [5.00 to 12.00]	5.00 [3.25 to 6.00]	0.01	1.02	2.50 (1.30 to 3.70)

Data are presented as mean ± SD or median (IQR [25th-75th percentiles]), as appropriate. 5STS: five-times sit-to-stand test; CG: control group; FSS-BR: Fatigue Severity Scale – Brazilian version; HGS: handgrip strength; mMRC: modified Medical Research Council Scale; PCG: post-COVID-19 group; PSQI: Pittsburgh Sleep Quality Index; SF-12: 12-Item Short Form Survey.

In the multiple linear regression analysis, considering PWV as the dependent variable and adjusting for sex, age, SBP, and DBP, the association between PWV and post-COVID-19 status was no longer significant (β = 0.24; 95%CI, −0.03 to 0.51; p = 0.08). In contrast, age (β = 0.117; 95%CI, 0.104 to 0.130; p < 0.001) and SBP (β = 0.032; 95%CI, 0.026 to 0.039; p < 0.001) remained independently associated with increased PWV.

In the subgroup analysis, individuals with arterial stiffness (n = 18) were significantly older and exhibited higher peripheral and central blood pressure levels, with moderate-to-large effect sizes. Conversely, no significant differences were observed in clinical-functional outcomes, including functional performance, fatigue, dyspnea, and quality of life, for which effect sizes were small and the 95%CIs included the null value (Table 4). Sex and DBP were not significantly associated with PWV.

**Table 4:** – Demographic, anthropometric, hemodynamic, and clinical-functional characteristics of post-COVID-19 patients with and without arterial stiffness

Variables	With arterial stiffness (n = 18)	Without arterial stiffness (n = 14)	p-value	Cohen’s d	Mean difference (95%CI)
**Age, years**	67.30 ± 5.10	49.60 ± 6.50	< 0.001	3.03	17.70 (13.50 to 21.90)
**Sex, M/F**	5/13	4/10	0.910	—	—
**BMI, kg/m**^**2**^	29.90 ± 4.30	33.50 ± 5.80	0.080	−0.70	−3.60 (−7.80 to 0.60)
**PWV, m/s**	9.84 ± 0.85	7.12 ± 0.64	< 0.001	3.60	2.72 (2.20 to 3.24)
**Peripheral SBP, mmHg**	140.30 ± 15.20	126.40 ± 11.90	0.010	1.01	13.90 (4.20 to 23.60)
**Peripheral DBP, mmHg**	89.50 ± 10.80	83.60 ± 8.90	0.040	0.59	5.90 (0.40 to 11.40)
**Central SBP, mmHg**	128.90 ± 14.10	116.80 ± 12.30	0.020	0.91	12.10 (3.00 to 21.20)
**Central DBP, mmHg**	90.20 ± 11.50	84.70 ± 9.40	0.040	0.53	5.50 (0.30 to 10.70)
Functional performance					
5STS, s	14.90 ± 4.20	16.30 ± 5.00	0.070	−0.01	−0.05 (−3.50 to 3.39)
HGS, kgf	32.60 ± 9.80	31.10 ± 8.70	0.220	−0.30	−1.40 (−4.80 to 2.00)
Quality of life, SF-12					
Physical component	39.70 ± 8.90	38.80 ± 9.10	0.330	0.16	1.50 (−5.30 to 8.30)
Mental component	41.20 ± 10.50	44.50 ± 11.20	0.290	−0.10	0.90 (−5.80 to 7.60)
**Fatigue, FSS-BR**	4.60 [3.30 to 5.30]	4.90 [4.20 to 5.70]	0.310	−0.30	−0.30 (−1.20 to 0.60)
**Dyspnea, mMRC**	3.00 [2.00 to 3.00]	2.00 [2.00 to 3.00]	0.410	−0.20	−0.62 (−1.71 to 0.46)
**Sleep quality, PSQI**	7.00 [5.00 to 10.00]	8.00 [6.00 to 12.00]	0.380	0.25	−1.00 (−0.50 to 2.50)

Data are presented as mean ± SD or median (IQR [25th-75th percentiles]), as appropriate. 5STS: five-times sit-to-stand test; BMI: body mass index; DBP: diastolic blood pressure; FSS-BR: Fatigue Severity Scale – Brazilian version; HGS: handgrip strength; mMRC: modified Medical Research Council Scale; PSQI: Pittsburgh Sleep Quality Index; PWV: pulse wave velocity; SBP: systolic blood pressure; SF-12: 12-Item Short Form Survey.

Central Illustration summarizes the main findings of the study, highlighting the increase in arterial stiffness and hemodynamic alterations, as well as the worsening of functional performance, fatigue, dyspnea, quality of life, and sleep quality observed in the PCG.

## Discussion

The findings of this study demonstrated that post-COVID-19 individuals exhibit significantly increased arterial stiffness, as evidenced by higher PWV and AIx@75 values, as well as alterations in central and peripheral hemodynamic parameters. Additionally, such individuals showed poorer functional performance, higher levels of fatigue and dyspnea, and impaired quality of life compared with controls matched for age, sex, BMI, and comorbidities. Collectively, these findings suggest the persistence of cardiovascular and clinical-functional alterations following SARS-CoV-2 infection, with potential medium- and long-term implications.

Although the age range was defined to reflect the patient profile of the pulmonology service where the study was conducted, most participants were older than 60 years and had overweight or obesity. This finding is consistent with prior research identifying advanced age and obesity as important risk factors for SARS-CoV-2 infection and for the development of severe forms of the disease.^21,22^

Although not directly assessed, the psychological impact associated with COVID-19 became evident during recruitment. Of the 103 eligible patients, 71 declined participation, reporting that they did not wish to revisit traumatic experiences related to the disease or family losses resulting from the pandemic. This finding is consistent with evidence demonstrating a high prevalence of emotional distress, psychological disorders, and trauma-related manifestations among COVID-19 survivors.^23,24^

Regarding medication use, there was a predominance of drugs prescribed for the management of stable clinical conditions, including antihypertensive agents for controlled systemic arterial hypertension (e.g., angiotensin-converting enzyme inhibitors, angiotensin II receptor blockers, calcium channel blockers, and diuretics), statins, and psychotropic medications (e.g., selective serotonin reuptake inhibitors and anxiolytics), with no significant differences between groups. Therefore, the influence of medications on the arterial stiffness and hemodynamic parameters assessed was likely limited, reducing the possibility of substantial confounding bias.

The vascular alterations observed may be related to pathophysiological mechanisms described in COVID-19, including persistent endothelial dysfunction, chronic inflammation, coagulation activation, and dysregulation of the renin-angiotensin-aldosterone system.^25-27^ When combined with preexisting comorbidities such as hypertension and obesity, these alterations may further increase medium- and long-term cardiovascular risk.^28,29^

The association between increased arterial stiffness and poorer functional performance observed in this study suggests a potential role of vascular dysfunction in the functional limitations experienced by these patients. Reduced arterial compliance may impair tissue perfusion during exertion, contributing to greater perceptions of fatigue and dyspnea, even in the absence of established cardiovascular disease.

In the current study, individuals in the PCG exhibited increased arterial stiffness accompanied by higher peripheral and central SBP levels. Given the close pathophysiological relationship between blood pressure and arterial stiffness, it is not possible to determine whether increased PWV contributed to higher blood pressure levels, whether blood pressure influenced PWV values, or whether both share common pathophysiological determinants. This limitation is inherent to the cross-sectional design and highlights the need for longitudinal studies capable of clarifying the temporal direction of these associations.

Furthermore, in the multiple linear regression analysis adjusted for age, sex, and blood pressure levels, the association between post-COVID-19 status and PWV values was no longer statistically significant. In contrast, age and SBP remained independently associated with increased PWV. These findings suggest that the greater arterial stiffness observed in the PCG may be predominantly explained by hemodynamic factors and vascular aging rather than by an independent effect of post-COVID-19 condition.

This finding reinforces the multifactorial nature of arterial stiffness and is consistent with the literature recognizing age and blood pressure as its primary physiological determinants. In the context of COVID-19, although mechanisms such as endothelial dysfunction and persistent inflammation have been extensively described, the results of the present study suggest that these alterations may act as amplifying factors on a previously vulnerable vascular substrate rather than as independent determinants of arterial stiffness.

Additionally, the absence of an independent association after adjustment highlights the need for caution when interpreting analyses based on PWV categorization, given that the definition of arterial stiffness itself was established using this parameter. In this context, the use of PWV as a continuous variable may represent a more methodologically robust approach in future studies.

In this context, the subgroup analysis comparing post-COVID-19 patients with and without arterial stiffness (Table 4) proved particularly enlightening: despite the significantly higher age and elevated central and peripheral blood pressure levels observed in the subgroup with arterial stiffness, no significant differences in clinical-functional outcomes—including functional performance, fatigue, dyspnea, and quality of life—were identified between the subgroups; effect sizes were small, and 95% confidence intervals included the null value. These results should be interpreted with caution, given the small sample size of the subgroup analysis, which may have limited the statistical power to detect actual differences.

This apparent dissociation suggests that, although VOP is strongly associated with vascular aging and hemodynamic determinants, its isolated presence does not fully explain the functional impairment observed in the post-COVID-19 condition. Indeed, patients in the GpC group exhibited poorer functional performance, higher levels of fatigue and dyspnea, and worse quality of life compared to the GC group; these findings may be better explained by additional mechanisms—including persistent systemic inflammation, prolonged hospitalization, physical inactivity, loss of muscle mass, and musculoskeletal alterations—acting independently or synergistically with the observed vascular changes, rather than necessarily by arterial stiffness alone.^30,31^

Our findings are consistent with previous studies demonstrating persistent functional impairment following the acute phase of COVID-19. In a previously published cohort, participants were classified into four groups according to the degree of physical and mental impairment, with severe impairment observed in 21% of individuals and very severe impairment in 17%. Furthermore, 46.2% presented persistent functional impairment, even when assessed using instruments different from those employed in the present study.^32^

Similarly, Huang et al. followed patients for 12 months after infection and observed a higher prevalence of persistent symptoms compared with controls, including dyspnea, mobility limitations, anxiety, depression, and fatigue, particularly among women.^33^

Comparable findings were reported by Seeßle et al., who identified reduced exercise capacity, persistent fatigue, and sleep disturbances in more than half of the patients evaluated 12 months after infection.^34^ Other studies have also reported prolonged physical and mental impairments associated with greater disease severity during the acute phase of COVID-19.

Interventional studies have also demonstrated meaningful therapeutic benefits in this population. In a clinical trial conducted by Jimeno-Almazán et al., 82% of participants reported fatigue, 59% reported dyspnea, and 51.3% reported sleep disturbances. After eight weeks of supervised exercise training, significant improvements were observed in fatigue assessed by the FSS-BR, quality of life assessed by the SF-12, perceived dyspnea, HGS, and functional performance assessed by the 5STS.^35^

Consistently, structured rehabilitation programs have demonstrated benefits in functional capacity, muscle strength, fatigue, sleep quality, and dyspnea, reinforcing the importance of multidisciplinary therapeutic approaches for individuals with persistent symptoms following COVID-19.^36-38^

Considering the findings observed and the growing recognition of the clinical relevance of arterial stiffness as a cardiovascular marker, the importance of comprehensive and early assessment of post-COVID-19 patients, particularly those with cardiovascular risk factors, is reinforced. The identification of vascular, hemodynamic, functional, and psychosocial alterations may contribute to the development of targeted monitoring and rehabilitation strategies. Although arterial stiffness assessment remains relatively underutilized in Brazilian clinical practice, it is a noninvasive, reproducible, low-cost method with potential applicability in this setting.

### Study limitations

This study has several limitations. The cross-sectional design precludes causal inference between SARS-CoV-2 infection and the observed alterations, making it impossible to establish the temporal direction of these associations. In addition, potentially relevant variables related to arterial stiffness, such as lipid profile, dietary patterns, and family history of cardiovascular disease, were not assessed. The high refusal rate during recruitment may have introduced selection bias, as individuals with greater psychological burden may have been less likely to participate. Finally, although validated, the oscillometric device used does not correspond to the tonometric method considered the reference standard for arterial stiffness assessment.

On the other hand, to the best of the authors’ knowledge, few studies have simultaneously evaluated arterial stiffness, hemodynamic parameters, and clinical-functional outcomes in post-COVID-19 patients, which adds originality and relevance to the findings presented.

## Conclusion

The post-COVID-19 patients evaluated in this study exhibited increased arterial stiffness, significant alterations in AIx@75 and central and peripheral SBP, poorer functional performance, lower quality of life, higher levels of fatigue and dyspnea, and poorer sleep quality. These findings underscore the complexity of post-COVID-19 condition and highlight the need for longitudinal studies to better understand the underlying mechanisms. Furthermore, they emphasize the importance of cardiovascular and functional assessment in these patients, as well as the implementation of multidisciplinary therapeutic strategies aimed at their follow-up and rehabilitation.
